# The effect of mild dehydration on physical fitness of elderly individuals

**DOI:** 10.1186/2046-7648-4-S1-A59

**Published:** 2015-09-14

**Authors:** Maria-Vassiliki Andrianopoulou, Nickos Geladas, Maria Koskolou

**Affiliations:** 1 Department of Sports Medicine and Biology of Exercise, Faculty of Physical Education and Sport Science, University of Athens, Greece

## Introduction

The detrimental effect of dehydration on exercise performance has been studied extensively [1]. However, there is a scarce data regarding the importance of adequate hydration on the functional capacity of elderly individuals to carry out daily physical activities. In this study, the effect of mild dehydration on physical performance of elderly (60 to 75 years old) healthy subjects was evaluated.

## Methods

Ten male and ten female healthy participants, 65.5(4.7) years old, performed a fitness test battery especially developed for this population, the Senior Fitness Test [2], both euhydrated (HYD) and dehydrated (by 1 to 2% of body mass) (DEH), following a random and counterbalanced order. In DEH, participants had to abstain from fluid intake for 24 hrs, while in HYD they were encouraged to consume liquids ad libitum for 24 hrs (a minimum of 2.5 L.day^-1 ^was suggested) before exercise testing. Hydration level was assessed by means of urine specific gravity (USG) and urine color chart (UCC). A USG value ≥1020 was defined as DEH, while USG <1020 as HYD. In addition to the scores achieved in the physical fitness tests, heart rate, arterial pressure, body weight and thirst sensation were recorded at rest and during recovery.

## Results

A 1,4% loss of body mass (p < 0.001) was achieved on average in DEH and the perception of thirst was greater (p < 0.001). Lower scores compared with the HYD condition were observed in the tests: 6-min walking test; 6MWT [DEH: 521.3(79.4) m vs. HYD: 565.8(94.8) m; p < 0.001] and 30-sec chair sit-to-stand [DEH: 17.9(5.1) rpts vs. HYD: 19.5(4.1) rpts; p < 0.05]. No significant difference (p > 0.05) was found between DEH and HYD in the tests: arm curl [DEH: 24.9(3.9) rpts vs. HYD: 26.5(5.8) rpts; p = 0.101], back scratch [DEH: -9.2(9,3) cm vs. HYD: -8.0(9.4) cm; p = 0.119], chair sit and reach [DEH: 0.0(9.1) cm vs. HYD: 1(10.2) cm; p = 0.118], 8-feet trial up and go [DEH: 5.0(1.1) sec vs. HYD: 4.9(1.1) sec; p = 0.119]. Males and females did not differ in performance of the fitness tests and no significant interaction was detected between gender and hydration status in any test (p > 0.05).

## Discussion

It was shown that even a mild dehydration corresponding to 1.4% of body mass was adequate to impair performance of lower extremities, as evaluated by the sit-to-stand test, and exercise tolerance, as judged by the 6MWT, in elderly people. The distance covered in the 6MWT is an index of cardiopulmonary endurance and reflects the functional exercise level required for daily activities in elderly individuals, since most activities of daily living are performed at submaximal level of exertion [3].

## Conclusion

The results of the present study underline the importance of adequate hydration on a daily basis for preserving functional capacity and exercise tolerance in old age.
